# Salinity and Bacterial Diversity: To What Extent Does the Concentration of Salt Affect the Bacterial Community in a Saline Soil?

**DOI:** 10.1371/journal.pone.0106662

**Published:** 2014-09-04

**Authors:** Loredana Canfora, Giovanni Bacci, Flavia Pinzari, Giuseppe Lo Papa, Carmelo Dazzi, Anna Benedetti

**Affiliations:** 1 Consiglio per la Ricerca e la Sperimentazione in Agricoltura, Centro di Ricerca per lo studio delle relazioni tra Pianta e Suolo, Roma, Italy; 2 Dipartimento di Scienze Agrarie e Forestali, Università degli Studi di Palermo, Palermo, Italy; 3 Department of Biology, University of Florence, Florence, Italy; U. S. Salinity Lab, United States of America

## Abstract

In this study, the evaluation of soil characteristics was coupled with a pyrosequencing analysis of the V2-V3 16S rRNA gene region in order to investigate the bacterial community structure and diversity in the A horizon of a natural saline soil located in Sicily (Italy). The main aim of the research was to assess the organisation and diversity of microbial taxa using a spatial scale that revealed physical and chemical heterogeneity of the habitat under investigation. The results provided information on the type of distribution of different bacterial groups as a function of spatial gradients of soil salinity and pH. The analysis of bacterial 16S rRNA showed differences in bacterial composition and diversity due to a variable salt concentration in the soil. The bacterial community showed a statistically significant spatial variability. Some bacterial phyla appeared spread in the whole area, whatever the salinity gradient. It emerged therefore that a patchy saline soil can not contain just a single microbial community selected to withstand extreme osmotic phenomena, but many communities that can be variously correlated to one or more environmental parameters. Sequences have been deposited to the SRA database and can be accessed on ID Project PRJNA241061.

## Introduction

Saline soils are environments characterised by high concentrations of salts and by an uneven temporal and spatial water distribution. According to an early classification, a soil is considered to be saline when the Electrical Conductivity (ECe) of a saturated paste is greater than 4 dS m^−1^ [1]. More recently, the two international soil classification systems introduced higher minimum thresholds of ECe to classify a soil horizon as saline. In fact, the WRB (World Soil Resources Reports) [2] considers the reference value 15 dS m^−1^ of ECe in defining the saline horizon, while the Soil Taxonomy [3] fixes the threshold at 30 dS m^−1^. A high concentration of salt in soil changes the availability of water and nutrients for both plants and microorganisms, and it has direct and indirect influences on soil organic matter, content, and stability [4]. Salinity, in fact, has been found to influence the size and the activity of soil microbial biomass [5], [6], which in turns plays a key role in biogeochemical cycles.

A basic distinction must be made between primary and secondary salinisation processes. Primary salinisation consists of salt accumulation through natural processes, such as a high salt content of the parent material or in groundwater. Secondary salinisation is usually caused by human interventions such as inappropriate irrigation practices, i.e. after the use of salt-rich irrigation water, and/or insufficient drainage. A natural secondary soil salinisation mechanism is represented by the long-term effects of tsunami waves, which can deposit salty seawater on large flooded areas with dramatic consequences for agriculture. Depending on the climatic conditions, secondary soil salinisation can also be temporary, and the soils may recover by washing out the infiltrated salt deposits through rainfall; but this is not the case for Mediterranean and arid environments, which can hardly spontaneously recover from a secondary salinisation event. Microorganisms that occur in naturally saline habitats are supposed to share a strategy for resisting high salt concentrations, and to have developed multiple adaptations for maintaining their population active while coping with such extreme environmental conditions. From the genetic point of view, these species display an under- or over-expression of peculiar genes and metabolites, which confer them the capability of coping with an osmotic stress [7].

A naturally saline soil is also a mutable environment where rain and water movements can strongly change the distribution of salts, and create an evolving patchy landscape. The researcher's perception of environmental variability, and, consequently, the scale at which specific properties (like salinity) are measured, can misrepresent the spatial scale at which microbial groups differentiate their structure. Measurements of microbial community's structure and function are often based on broad-scale characterisations, and rarely consider the real spatial scale within which individuals and populations interact [8], [9]. Space and scale in population, community, and ecosystem processes are increasingly recognised as fundamental factors in the study of microbial functions and activities in soil [8]. Understanding the spatial pattern in the abundance and structure of microbial communities occurring in saline soils represents a crucial target in ecology [9], [10] as it sheds light on the selection mechanisms exerted by the environment on bacterial groups with specific functions and properties. The choice between a niche higher in salinity and instability, where instead of developing metabolic tools for resistance the bacterial communities wait for more favorable conditions, can represent the key mechanism that shapes microbial heterogeneity and taxa spatial composition in problematic soils.

In this framework, a series of surveys on “extreme“ environments where salinity is the main determinant addressed the issue of some microbial taxa's specific ability to resist osmotic stress or other limiting factors associated with the presence of high salt concentrations. On the basis of these studies [11], [12], [13], [14], [15], [16], [17], [18], [19], [20], [21], [22], [23], [24], [25], [26], [27], [28], we have attempted a rough discrimination between taxa frequently listed in these extreme environments (salinity related), and taxa less represented or even absent in salt affected sites (salinity unrelated). Clearly, this approach can lead to a dangerous generalisation, especially for the taxa that contain genera and species with strongly different physiologies and very broad geographical distributions. This is the case of the phylum *Proteobacteria*, which is probably the biggest group of bacteria associated to practically any environment.

A recent meta-analysis of soil microbial communities reported that the global microbial composition in a saline soil is influenced more by salinity than by any other extreme chemical factors such as temperature or pH [11], [12]. Ma and Gong [12] recently examined the bacterial and archaeal diversity in saline soils using a meta-analysis approach indicating that approximately 50% of the archaeal diversity and less than 25% of the total bacterial diversity has been recorded from saline soil habitats. Ma and Gong [12] updated the available information on DNA sequences gained from a wide array of studies on soil microorganisms in saline environments, but also showed that there is a significant gap in the published information on the relevant soil properties where microbial communities had been sampled. Despite the useful and unique information collected, the authors could not address the beta diversity of the microbial species according to the different salt concentrations [12], thus failing to relate bacterial taxa composition with such drastic soil physical-chemical factors.

In the present study, a thorough evaluation of soil characteristics was supported by a pyrosequencing of the V2–V3 16S rRNA gene region in order to investigate the bacterial structure and diversity in the A horizon of a natural saline soil habitat located in Sicily (Italy). The application of metagenomic strategies has been recognised by several authors as a valid instrument to exploit the microbial biodiversity within soil habitats [29], [30], [31], but up to now saline habitats have still been largely unexplored by the metagenomic approach. The main aim of the research is to evaluate the organisation and diversity of microbial taxa by means of a spatial scale that reveals heterogeneity in the distribution of physical and chemical properties of the environment under investigation. As salinity is one of the most widespread soil degradation processes on earth affecting an estimated 1 million hectares in the European Union, mainly in the Mediterranean countries, and as it is a major cause of desertification, the understanding of soil microbial resilience and resistance in primary salinisation processes represents a cognitive platform for any application of bioremediation in secondary salinisation events.

## Materials and Methods

### Site description

The study was performed in Sicily, Italy, in an abandoned natural area, *Piana del Signore* -a semiarid Mediterranean environment- characterised by an alluvial flat land where the geomorphology has been modeled by the river *Gela* (Fig. S1). The area falls within the Natura 2000 network as a site of Community Importance (SCI), with the number SIC ITA050012- Gela. In the basin of the river *Gela* the prevalent lithology is made up by Messinian evaporites belonging to the *Gessoso-Solfifera* geological formation, among which many types of saline rocks crop out: Gypsum, Carbonates and Marls (with frequent chloride and sulfide rock inclusions). The area we surveyed is 12.3 hectares wide, and lies about 1 km far from the coastline and 1.2 km from the river estuary. The vegetation is a patchy mosaic plant association defined *Junco subulati-Salicornietum fruticosae* belonging to the *Thero-Salicornietea* class, in which the most plants are salt pioneer swards typical of salt marshes. The vegetation pattern consists of *Salicornia fruticosa* (L.), *Suaeda fruticosa* (L.), *Juncus subulatus* (Forsskal), *Juncus bufonius* (L.), *Phragmites australis* (Cav.), *Aster squamatus* (Spreng.), *Polypogon monspeliensis* (L.), *Hainardia cylindrica* (Wild.). The vegetation distribution is discontinuous: plant covered zones alternate with bare zones with visible salt crusts deposited above the soil surface. The whole area is temporarily flooded in the autumn and winter seasons, with a long permanence of water in those zones where salt crusts have formed on the soil surface after the water has dried out. Mean daily air temperature ranges from a maximum of 26.6°C in August to a minimum of 4.9°C in January. The average annual rainfall is 383 mm according to the nearest meteorological station (37°4′48″N, 14°13′12″E).

### Soil Survey and sampling

Nine soil sites from A horizons (with a mean depth of 0–10 cm) were collected following a random simple sampling scheme (Fig. S2a) in Summer 2011. No specific permission was required for this location, as no endangered or protected species live in the area (Table S1). Sampling sites were positioned with a minimum distance between points of 50 m. Sites were accurately recorded with a GPS. In each site, three soil samples were collected in the vertices of 1-meter side equilateral triangle (Fig. S2b) and mixed together in a unique representative analytical sample [32]. Vegetation, salt crusts, and other features of the soil surface were described and recorded for each site. Soil samples from each site were subdivided in two representative subsamples: the first one was air dried, 2 mm sieved, then chemically and physically analysed, while the second one was stored at −80°C and later processed for the 454 pyrosequencing analysis reported below.

### Soil physical and chemical analysis

Air-dried and sieved soil subsamples were analysed for the following physical and chemical properties: texture; reaction (pH), electrical conductivity (EC), and organic carbon (C_org_). Texture was determined by the pipette method, without carbonate and organic matter removal, and after complete removal of soluble salts by using distilled water [33]; pH was measured on 1∶2.5 (w/v) soil to water mixtures; EC_1∶5_ was measured on 1∶5 (w/v) soil to water mixtures at 25°C; C_org_ was obtained using the Walkley and Black method; EC_1∶5_ was converted in electrical conductivity of the saturation paste extract (EC_e_) using the correlation model proposed by Khorsandi and Yazdi [34] for arid and semiarid environments.

### DNA extraction

DNA was extracted from the fresh soil sub-sample with the MoBio Power soil DNA extraction kit following the manufacturer's instructions. The samples were then purified from excess impurities with GeneReleaser [35]. DNA crude extract concentrations were measured using Qubit 2.0 Fluorometer by means of the kit Quanti HS assay Invitrogen, following the manufacturer's instructions. DNA was extracted in duplicates from each soil site, and then pooled and used for the following analytical steps.

### Amplification of 16S rRNA genes and pyrosequencing

The V2–V3 region of the 16S rRNA bacterial gene was amplified by PCR. The PCR reaction mixture (50 µl) contained 10 µl 10-fold reaction buffer (Fusion GC buffer, FINNZYMES, Espoo, Finland), 800 mM of each of the four deoxynucleoside triphosphates, 3% DMSO, 1.2 mM of each of the primers, V2 For and V3 Rev, 0.5 U of Phusion hot start high- fidelity DNA Polymerase (FINNZYMES), and 20 ng of isolated DNA as template. The V2–V3 region was amplified with the following set of primers containing the Roche 454 pyrosequencing adaptors (underlined): V2for 5′-CGTATC**GCCTCCCTCGCGCCA** TCAG **ACGACTGCGTAGTGGCGGACGGGTGAGTAA**-3′ and V3rev 5′- CTATGC**GCCTTGCCAGCCCGC** TCAG **AGACGCACTCATTACCGCGGCTG**C-3′ ([31], modified following the instructions of BMR Genomics to better adapt the primers to the 454 project and overcome possible PCR biases. (In bold, the reader can find the starting bp number for each primer modified, adding the tag CGTATC and CTATGC at 5′ and a barcode in the middle of each primer TCAG). The following thermal cycling scheme was used: initial denaturation at 98°C for 5 min, 25 cycles of denaturation at 98°C for 45 s, annealing at 68°C for 45 s, and extension at 72°C for 25 s, followed by a final extension period at 72°C for 5 min. All samples were amplified in two series of triplicates, pooled in equal amounts, and then purified using the Qiaquick PCR purification kit (Qiagen Inc. Chatsworth, CA, USA). Six independent PCR products for each site were combined to minimise the impact of PCR errors. Quantification of the PCR products was performed using the Quant-iT dsDNA BR assay kit (Invitrogen GmbH, Karlsruhe, Germany) and a Qubit fluorometer (Invitrogen GmbH, Karlsruhe, Germany) as recommended by the manufacturer. The samples were stored at −20°C and sent to BMR Genomics s.r.l. (Padova, Italy) for pyrosequencing by means of a Genome Sequencer FLX System platform (454 Life Science Branford, CT, USA).

### Analysis of pyrosequencing data: dataset clean up and taxonomic assignments

The obtained sequences were assigned to each site using a custom script developed by BMR Genomics. 9 different sequence files, with an average sequence number of 6108, and an average length of 492 nucleotides per sequence were obtained (Table S2, Table S3). In order to ensure a correct nucleotide assignment in the raw sequence files, 2 control steps were performed. First, the nucleotide distribution along each sequence was analysed. This analysis showed that each file had an unbalanced nucleotide distribution in the first 5–10 bases of each sequence (Fig. S4). Next, the quality distribution along each sequence was analysed in order to identify possible low quality segments. As a result, low quality segments of 50–100 bases were identified in the terminal region of each sequence. Finally, a trimming step was performed using StreamingTrim software [36]. An offset of 10 nucleotides was set in order to remove the first 10 bases of each sequence. After the trimming step, 6062 sequences were collected with an average length of 425 bases.

The last control step performed consisted in the identification of chimeric sequences in the dataset [37]. In order to detect all possible chimeric sequences, a dataset was constructed that contained all the 16s rRNA available genes in the NCBI (National center for Biotechnology Information). This database was used as reference for the UCHIME algorithm [38]. After UCHIME analysis 1936 chimeric sequences were detected and removed from the dataset. As a result, 9 different sequence files (one for each site), containing an average sequence number of 5847, were recovered (for additional details, see Fig. S3).

In order to construct a community data matrix, the cleaned files were analysed using the standalone version of RDP multiclassifier [37]. As the average length of the sequences to be analysed was bigger than 250 nucleotides, an assignment's confidence cutoff of 0.8 was set to perform a much stringent analysis (according to the RDP multiclassifier pipeline: http://rdp.cme.msu.edu/tutorials/classifier/RDPtutorial_MULTICLASSIFIER.html).

### Statistical analysis of community data

The community data matrix obtained was used in a series of statistical and ecological evaluations carried out using the R software (http://cran.r-project.org/) [39] and the *vegan* package (http://cran.r-project.org/web/packages/vegan/index.html) [40]. A Rarefaction Analysis [41], based on *genus*-level data (all taxonomic assignments that reached the *genus* level), was performed in order to inspect the different grade of diversity explained in each site. *Richness* [42], *inverse Simpson* [43] and *evenness* [44] indexes were calculated on the same data set. *Richness* index was calculated on the basis of the number of the taxonomic assignments at the *genus* level gained for each site, while *evenness* and *inverse Simpson* were calculated as follows:
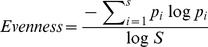


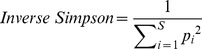



Where, S is the site *richness* (defined as the number of genera in each site) and p_i_ is the proportion of genera in the site [42]. In order to inspect the putative number of unseen *genera* present in the sites, Chao [45] has calculated the index using the following equation:
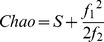



Where, S is the site *richness* (defined above), f_1_ and f_2_ are the numbers of genera observed once and twice respectively. The community data matrix has then been transformed into a relative abundance matrix in order to highlight the differences in the community composition correlated to the salinity and pH levels of each site. The relative abundance values have been calculated dividing the number of reads assigned to each *genus* by the total number of reads present in each sequence file, in order to compare these values with each others. Moreover, a Canonical Correlation Analysis (CCA) was run with soil salinity and pH fitted onto the ordination analysis obtained, using the *envfit* function of the *vegan* R package, with a number of permutations of 10000. To emphasise the differences in the composition of the different *taxa* inside the community data matrix, a relative abundance matrix was generated, as described above, considering just the assignments at the Phylum level. This matrix was used to generate a heatmap plot of the ordered sites, following the soil degree of salinity (from the low salinity level, at the bottom, to the high salinity level, at the top). Above the generated heatmap, a coloured bar was placed to indicate the phyla correlated to a high level of salinity and the phyla not strictly correlated to high salinity levels. In addition, a dendrogram showing a cluster distribution was plotted and added to the map, using the Bray-Curtis distance and the UPGMA algorithm.

Furthermore, data on soil properties and on the abundance of microbial phyla were combined as variables in a principal component analysis (PCA), used as exploratory analysis, assuming that microbial groups (absolute abundance values) have a linear response to environmental gradients [46]. The PCA was performed on autoscaled data, and based on Spearman's rank correlation matrix; XLStat 7.0 (Addinsoft, Paris) statistical software was used for the purpose. Spearman's rank correlation test was used to define the degree of dependence among the variables. The advantage of using Spearman's rank correlation coefficient is the independence of the population's distribution, so that the data can be collected over regular spaced intervals. The Spearman's correlation coefficient (rho) was used as a measure of the correlation (dependence) between the variables, giving a value between +1 and −1, where 1 stands for the total positive correlation, 0 stands for no correlation, and −1 stands for the total negative correlation. The rho coefficient is based on the ranks of the observations; the Spearman's rank correlation coefficient does not assume that the relationship among the variables is linear [47].

## Results

The 9 soil sites represented nine plots distributed in an area characterised by a great spatial variability, which encompassed different levels of salinity, and a significant variation in soil pH, organic carbon, vegetation type and cover percentage, saline crust percentage, and texture class (Table 1). Electrical conductivity ranged between 169.96 dS m^−1^ to 5.37 dS m^−1^ with values of pH between 6.4 and 8. Total organic Carbon ranged between 4.26 g kg^−1^ and 0.38 g kg^−1^.

**Table 1:** Main physical and chemical properties of the soil sites and percentage of vegetation and salt crust cover of the sampling sites estimated in the field.

Plot	pH	EC_e_	C_org_	Clay	Silt	Sand	Texture class^1^	Vegetation cover	Salt crust cover
		dS m^−1^	g kg^−1^					%	
**1**	7.9	50.45	2.27	189	177	634	loam	90	70
**2**	7.0	15.06	1.32	80	98	822	loamy sand	85	5
**3**	7.2	92.73	0.67	76	74	850	loamy sand	40	100
**4**	7.4	28.00	4.26	245	294	461	silty loam	100	0
**5**	7.8	5.82	1.4	83	145	772	loamy sand	100	0
**6**	6.9	63.50	1.46	369	263	368	silty clay loam	90	0
**7**	7.2	169.96	2.35	188	217	595	loam	3	100
**8**	8.0	5.37	0.38	54	38	908	sand	70	0
**9**	7.5	54.88	1.27	107	105	788	sandy loam	90	100

1 Classification according to the international scheme (Leeper and Uren, 1993).

Microbial DNA extraction yielded between 4.5 ng/µl to 10.2 ng/µl, showing variability in DNA recovery. Given the very standardised methodology applied to all the samples during the extraction step, the reason for the yield variability can be searched in the different soil characteristics among the nine sites. A different salt concentration and the presence of a variable amount of organic carbon can modify the degree of interaction between microbial DNA and the used extracts [48]. In order to check whether possible interferences occur between the DNA extraction procedure here adopted and some of the main soil properties, namely Organic Carbon content, pH and Salt concentration, an evaluation of the degree of correlation (Spearman's correlation) between DNA recovery (ng/µl) and measured soil parameters has been carried out. The results (Table S5) excluded a statistically significant positive or negative role of soil organic carbon, pH and salt concentration in DNA recovery during extraction.

### General analyses of the pyrosequencing- derived dataset

**Figure 1:**
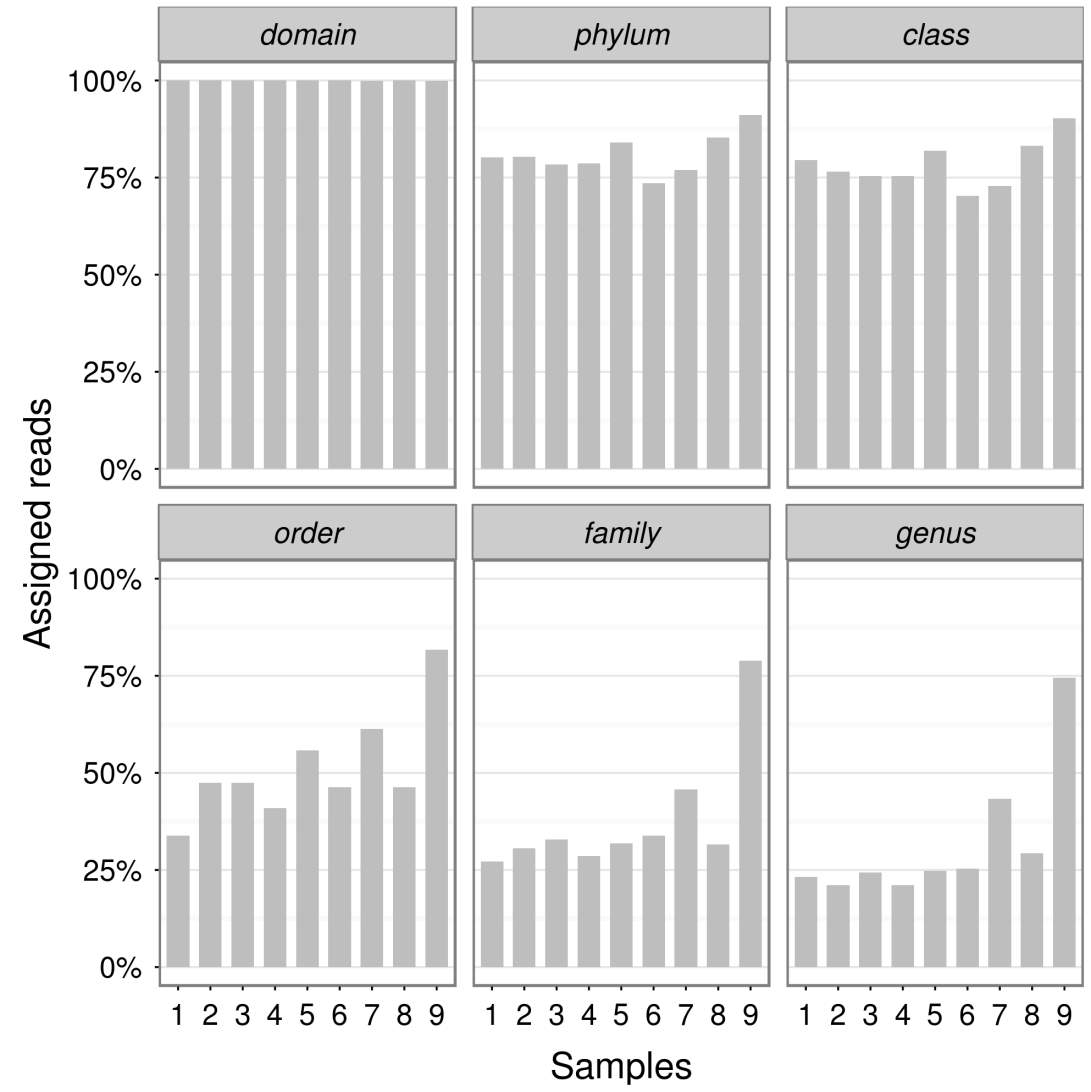
Number of sequences assigned by RDP multiclassifier for each site at each taxonomic level.

The average length of the sequences analysed was bigger than 250 nucleotides, more than 75% of the reads analysed was assigned at the Phylum level, and just 25–30% of the reads was assigned to the *genus* level. The description of the assignments' number at each taxonomic level and for each site is reported in Figure 1. A total of 16342 sequences for the nine soil sites was used for bacterial diversity analysis. Figure S4 displays the nucleotide's relative frequency distribution along the sequences, where the first 10 bases of each sequence file have an unbalanced nucleotide distribution, suggesting an uneven distribution of sequences among the sites in the soil. The number of sequences per site ranged from 1968 to 15614, except for site 1. Figure 1 displays the number of sequences assigned by RDP classifier for each site above and below the domain of Bacteria 52617 sequences were assigned to the domain of Bacteria and 42459 of these sequences were classified below the domain level.

### Bacterial diversity and richness

**Figure 2:**
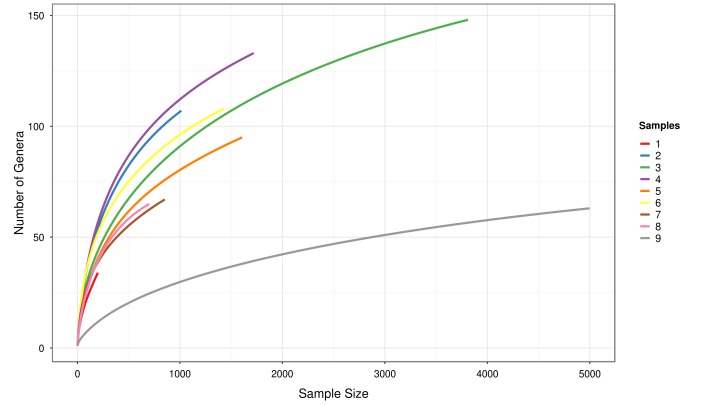
Rarefaction curves of the sites. These curves were obtained considering only the taxonomic assignments that reached the *genus* level in the RDP multiclassifier analysis.

Figure 2 shows the rarefaction curves obtained for each soil site. Rarefaction curves were created by randomly re-sampling the pool of N samples multiple times, and then plotting the average number of species found in each site. This method generated the expected number of species in a small collection of n samples drawn at random from the large pool of N samples. Rarefaction curves grow rapidly at first, as the most common species are found, then the curves reach a plateau when the rare species remain to be sampled. In sites 1, 7 and 8 the curves didn't reach saturation, suggesting that taxonomic diversity was not fully exploited. In contrast, the remaining six sites showed rarefaction curves that reached a plateau. In particular, site 9 reached the point of saturation faster than the other sites.

**Figure 3:**
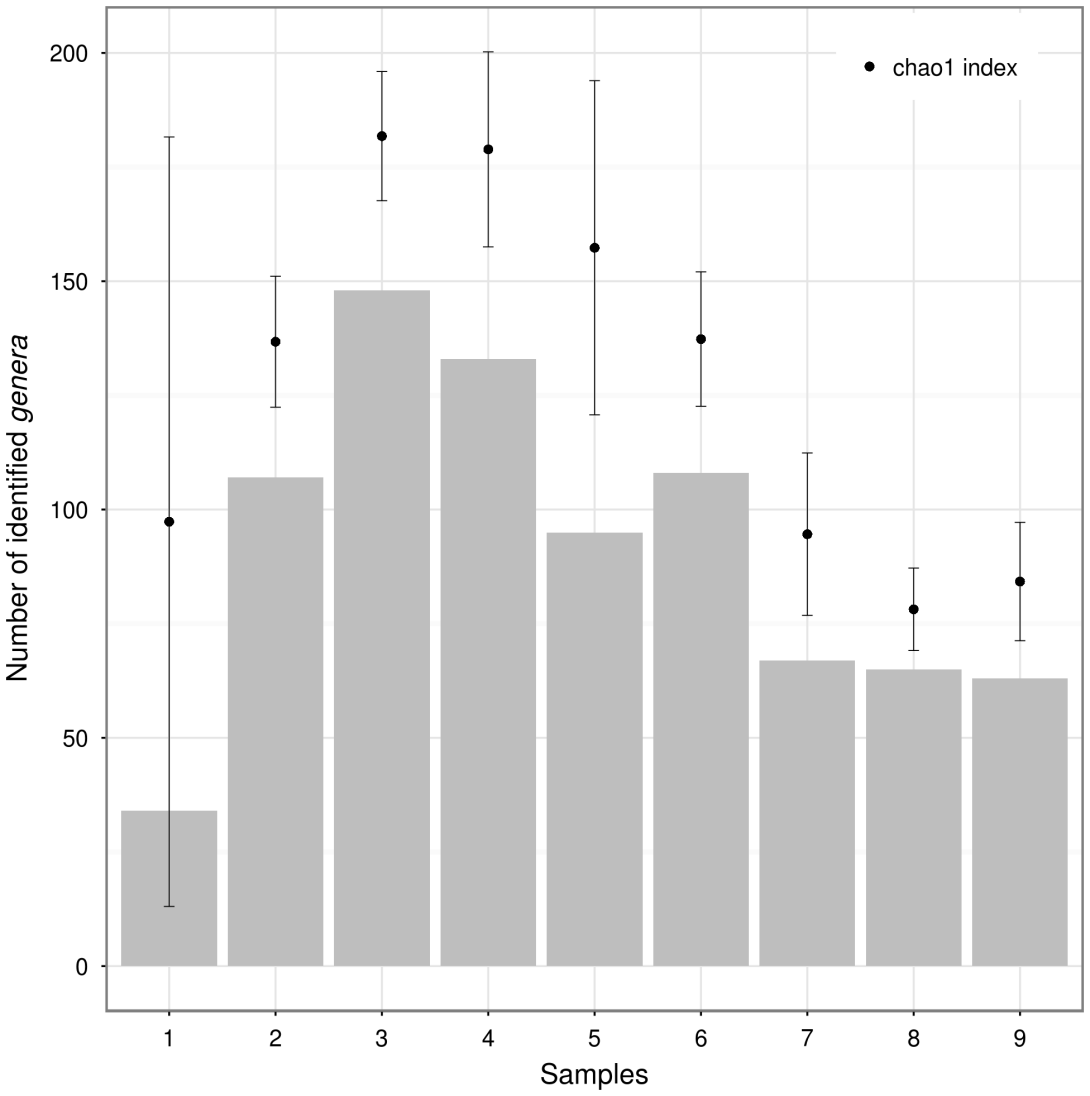
Richness values compared to Chao1 indexes. As shown in this plot almost all sites have a relevant number of putative “unseen *genera*” according to the Rarefaction analysis (Fig. 3).

A comparison of the rarefaction analyses with the Chao1 (Fig. 3) index revealed that a substantial fraction of soil sites (6 out of 9) showed a relevant number of putative “unseen genera”, in agreement with the rarefaction analysis.

In table 2, the values of biodiversity indexes calculated on the nine soil sites were compared; site 9, despite showing a rarefaction curve that reached the plateau, was characterised by low values of the *Invsimpson* and *evenness* indexes, when compared with the other soil sites. The values of the *Invsimpson* index (Table 2) ranged between 1.6 and 22.7 while the *richness* index ranged between 34 and 148. The *evenness* index ranged from 0.2 to 0.8, indicating an uneven distribution of bacterial genera between the sites.

**Table 2:** Diversity indexes. Each index has been calculated as reported in the section: “Statistical analysis of community data” of the “Material and Methods” chapter.

		value of biodiversity indices		
Plot	*Invsimpson*		*Richness*	*Evennes*
**1**	6.67		34	0.70
**2**	14.78		107	0.74
**3**	13.72		148	0.66
**4**	11.04		133	0.70
**5**	5.17		95	0.58
**6**	22.71		108	0.77
**7**	14.22		67	0.74
**8**	6.41		65	0.65
**9**	1.57		63	0.21

The comparison of the soil sites based on the values of the *richness* index showed the greatest bacterial richness in site 3, followed by sites 4, 6, and 2. Both *Invsimpson* and *richness* indexes (Table 2) discriminated between the different soil sites, but didn't succeed in emphasising peculiar behaviours of some sites. In the case of site 9, for example, which showed a curve of rarefaction very different from the other sites, the *evenness* index was the only indicator that showed sensitivity to this particular behaviour.

### Bacterial phyla distribution along a salinity gradient

The 52623 sequences classified below the domain level were affiliated to 15 bacterial phyla. The dominant phyla across all sites were: *Acidobacteria*, *Actinobacteria*, *Bacteroidetes*, *BRC1*, *Chlorobi*, *Chloroflexi*, *Cyanobacteria*, *Deferribacteres*, *Firmicutes*, *Gemmatimonadates*, *Nitrospira*, *Planctomycetes*, *Proteobacteria*, *Spirochaetes*, *Tenericutes*, *Verrucomicrobia*, *WS3*. The dominant taxa in the analysed soil sites were: *Proteobacteria* (95.95%), *Actinobacteria* (85.39%), *Acidobacteria* (72.12%), *Verrucomicrobia* (70.60%), *Firmicutes* (64.14%), followed by a second group showing a lower but still important percentage of distribution across all the soil sites (*Bacteroidetes*, *Chloroflexi*, *Chlorobi*, *Gemmatomonadates*, with a percentage between 70 and 50%). The taxa with a lower relative distribution were: *Planctomycetes*, *Tenericutes*, *Deferribacteres*, *Cyanobacteria*, *Spirochaetes*, *Nitrospira*, and the uncultured candidate bacterium divisions *WS3* and *BRC1*.

**Figure 4:**
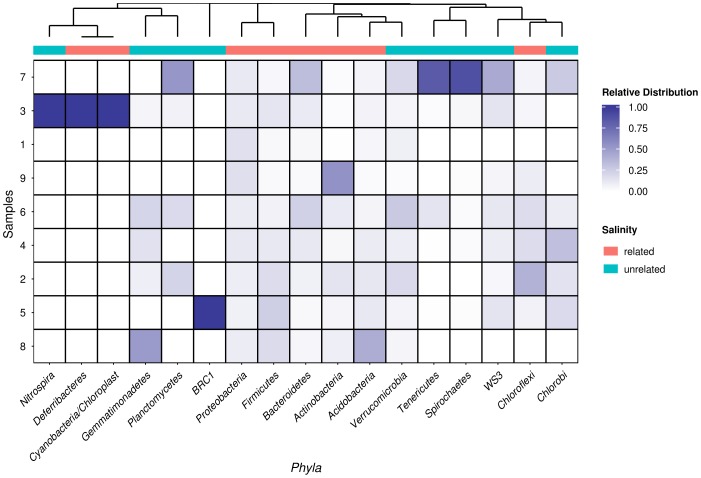
Heatmap of *Phyla* assignments. The heatmap reports the normalized values of the taxonomic assignments at *phylum* level. Each value has been normalized following this criterion: 
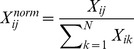
 Where *X_ij_* is the occurrence of the phyla ‘j’ in the site ‘i’ and N is the number of site in the dataset (in this case 9). Using this transformation each *phyla* assignment can be compared in all sites independently from its order of magnitude.

The relative distribution of the groups of bacteria varied among soil sites and along a salinity gradient, as showed in the heatmap of Figure 4, where the relative distribution of *phyla* assignments is clustered and plotted with respect to the different values of salinity of soil sites. Each row (sites) of the heatmap shows the phyla's relative abundance in a soil site. The rows of the heatmap are ordered according to the degree of salinity in the soil sites (from the lowest values, at the *bottom*, to the higher, at the *top*). The heatmap of Figure 4 reports the following annotations: “salinity related” and “ salinity unrelated”, referring to taxa frequently listed in saline environments and taxa less represented or even absent in salt affected sites, as roughly determined on the basis of what reported in previous studies [12], [13], [14], [15], [16], [17], [18], [19], [20], [21], [22], [23], [24], [25], [26], [27], [28]. Moreover, the cluster structure shows five main groups of phyla which share a peculiar composition and abundance among the sites. For example, site 3 presented a very peculiar abundance of three phyla (*Cyanobacteria*, *Deferribacteres*, *Nitrospira*), which resulted unrelated to salinity, while a large group of phyla, that appeared grouped together and uniformly distributed across the other sites, resulted strongly correlated to salinity, although showing a low abundance. In fact, the phyla *Acidobacteria*, *Actinobacteria, Firmicutes*, *Proteobacteria*, and *Bacteriodetes* resulted related to all the 9 sites, with almost the same abundance degree. *Spirochaetes* and *Tenericutes* resulted related just to site 7, and the BRC1 candidate Phylum was only found in site 5.

**Figure 5:**
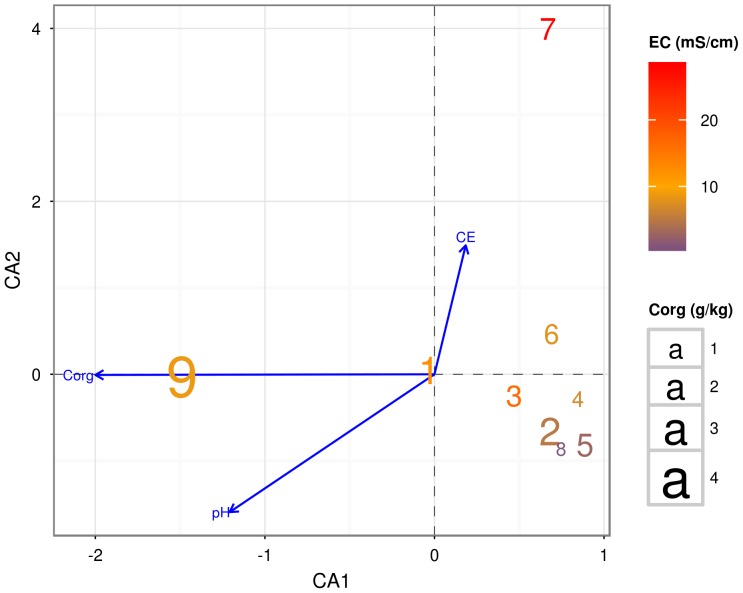
Canonical Correlation Analysis (CCA) based on community data matrix. The salinity levels and the pH levels of each site have been fitted onto the CCA ordination analysis in order to inspect the relevance of these two factors in relation to the bacterial communities distribution.

Salinity and pH values of each soil site were used as variables in the CCA ordination analysis, run in order to highlight the influence of these two soil properties on the bacterial community structure, and to highlight the phyla's relative abundance among sites (Fig. 5). The Canonical Correlation Analysis (CCA) (Fig. 5) summarises the joint variation of the two sets of variables, namely soil pH and salinity, in relation to the bacterial phyla ordination (obtained using the *envfit* function of the *vegan* R package, as described in the Materials and Methods Section). The CCA method combines a correspondence analysis with a multivariate regression analysis, taking into account the underlying model that assumes chi-squared dissimilarities among the sites. The plot obtained by CCA allowed us to extract synthetic environmental gradients from the metagenomic data-sets. The gradients are fundamental for succinctly describing and visualising the different habitat preferences (niches) of the phyla; in order to do so we used an ordination diagram that is shown in Figure 5. The graph obtained on the basis of permutations gave a clear picture of the variability expressed by the different sites, showing different ecological niches for both salinity and pH, but also with respect to the inhabiting microbial communities. In particular, Figure 5 showed a different spatial position within the plot of the sites 7, 9 and 1, due to a high salinity level in the first one, and due to a significantly lower pH in the other two sites. It also emerged that the bacterial communities of sites 5 and 8 were characterised by a tendency to low values of salinity, and that there was a core of sites with closer bacterial assemblages and soil characteristics (sites 2, 3, 4, 5, 6 and 8).

Spearman's rank correlation results (Table 3) show the dependence between a relative abundance of bacterial phyla and the soil properties. *Proteobacteria*, *Spirochaetes*, *Tenericutes*, *WS3 Plantomycetes* and *Bacteroidetes* showed a significant positive correlation with salinity. The *Acidobacteria* showed a statistically significant negative correlation with salinity (−0.717 for p<0.01). The only bacterial group that showed a relative abundance significantly correlated with soil pH was the *Plantomycetes*, which resulted distributed among sites with a negative correlation to the variable's values (a higher abundance of bacteria was related to lower pH values). Two phyla showed a statistically significant dependence on the organic carbon content (Corg) of soil sites: *Verrucomicrobia* and *Chlorobi*. Interestingly, the Spearman's rank correlation between the degree of salt crust coverage of the sites and the composition of bacterial phyla did not follow the trend showed by the variable ECe (salinity) and, while not showing statistically significant values, it divided the groups of bacteria into two categories, positively dependent and negatively dependent on the presence of a salt crust in the site.

**Table 3:** Spearman's rank correlations between the relative abundances of the six most abundant bacterial phyla and the soil properties across the nine soil sites.

Taxonomic group	Correlation values				
	ECe dS m−1	pH	Corg	Salt Crust cover	Vegetation Cover
***Proteobacteria***	0.500	0,142	0.317	0.541	0.034
***Actinobacteria***	−0.317	−0.226	−0.467	−0.213	0.196
***Acidobacteria***	−0.717**	0.084	−0.333	−0.638	0.009
***Verrucomicrobia***	0.217	−0.603	0.667**	−0.301	−0.17
***Firmicutes***	−0.650	0.109	−0.367	−0.585	0.051
***Bacteroidetes***	0.533	−0.661	0.467	−0.027	−0.400
***Chloroflexi***	−0.033	−0.655	0.301	−0.236	0.393
***Chlorobi***	0	−0.345	0.722*	−0.319	0.276
***Gemmatimonadetes***	−0.331	−0.179	−0.235	−0.625	−0.049
***WS3***	0.536	−0.445	0.301	0.183	−0.103
***Plantomyccetes***	0.566	−0.816**	0.146	0.330	−0.634
***Tenericutes***	0.688*	−0.586	0.444	0.079	−0.231
***Defferibacteres***	0.411	−0.206	−0.411	0.437	−0.420
***Cyanobacteria***	0.411	−0.206	−0.411	0.437	−0.420
***Spirochaetes***	0.712*	−0.468	0.390	0.271	−0.208
***Nitrospira***	0.411	−0.206	−0.411	0.437	−0.420
***BRC1***	−0.411	0.275	0	−0.364	0.490

In the table is reported the rho value. The significant correlation values are indicated as follows: * P<0.05; ** P 0.01.

**Figure 6:**
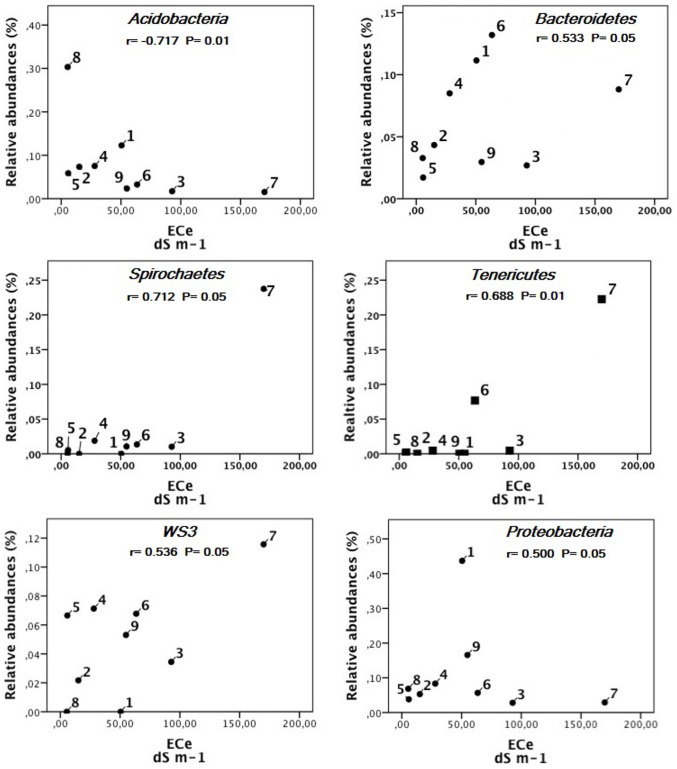
Correlations between relative abundances of different taxonomic groups and soil salinity. Circles represent the soil sites. Spearman rank correlation coefficient (r) with the related P values are shown for each taxonomic group.

Figure 6 shows the scatter plots of sites obtained by plotting the variables ”abundance“ and ”soil salinity (ECe) for each bacterial phyla. The plots allow us to check for linearity, that is, for a monotonic relationship between the two variables, and to check whether either the variables increase in value together, or, as one variable value increases, the other variable value decreases. The plots showed in Figure 6 relate to the bacterial phyla showing a significant rho value in the Spearman's rank correlation test. With regards to *Bacteroidetes*, *Spirochaetes* and *Tenericutes,* their relative abundances in the different sites increase positively with the salinity degree in the soil (from site 8, showing one of the lowest values of salinity, through to sites 5, 2, 4, 1, 9, until the sites 6,3, and 7 showing the highest values of salinity). *Proteobacteria* and *WS3* also showed a monotonic relationship between abundances and soil salinity, although some sites (i.e. site 1 for *Proteobacteria*) showed a non-linear behaviour. Furthermore, *Acidobacteria* showed an opposite trend, as relative abundances decrease with the increase of soil salinity, as also indicated by the Spearman's rank correlation test (Table 3).

**Figure 7:**
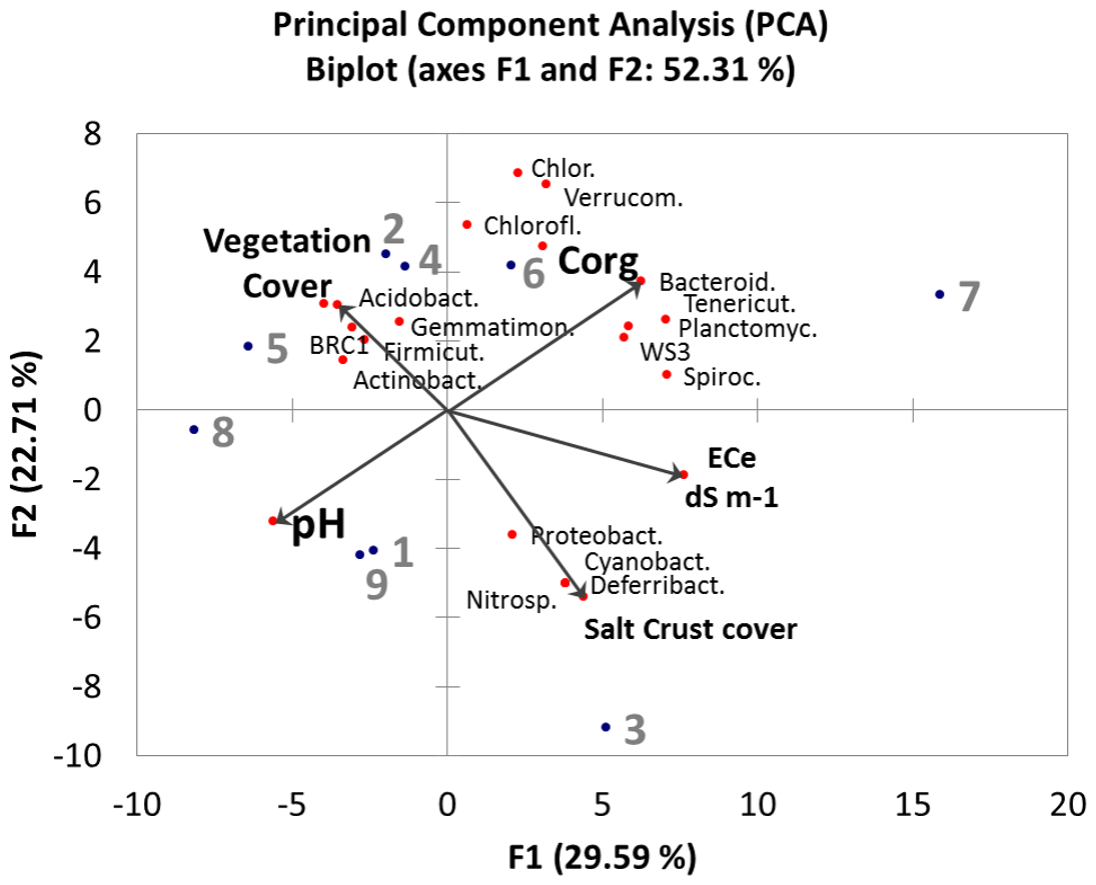
Principal component analysis of bacterial communities as affected by soil properties, based on the abundance of bacterial phyla. Every vector points to the direction of increase for a given variable so that soil sites with similar bacterial communities, are localized in similar positions in the diagram.

A principal component analysis is reported as biplot in Figure 7. The bacterial phyla abundances in each sample were used as variables, together with soil chemical properties (C_org_, pH, vegetation cover, salt crust, ECe), while the soil sites were showed as observations.

The biplot, obtained using the first two components, which together explained about 52.31% of the total variability of data, showed that salinity (ECe variable) is correlated with salt crust presence, and that both variables are positively correlated with the values of PC1 axes. Soil pH appeared correlated to the vegetation cover, and both variables are negatively correlated to the PC1 axes. Soil organic matter content (C_org_) is positively correlated to PC2 axes. The loading of the sites on the principal components axes confirms the main patterns that were delineated by both Spearman's rank correlation analysis (Fig. 6; Table 3) and the cluster analysis (Fig. 4). The variability of soil properties between the 9 sites corresponded to a diverse relative abundance of bacterial phyla, which actually showed a different degree of correlation with soil characteristics. In some cases, there is an inverse relationship between bacterial phyla, thus showing a kind of vicariousness, suggesting that the presence of some groups of bacteria in a site could be the reason for the absence of others. Rather the opposite behaviour is found in some phyla that are strongly associated in the biplot, and seem to occur in the same sites and when the same micro-environmental conditions occur.

**Figure 8:**
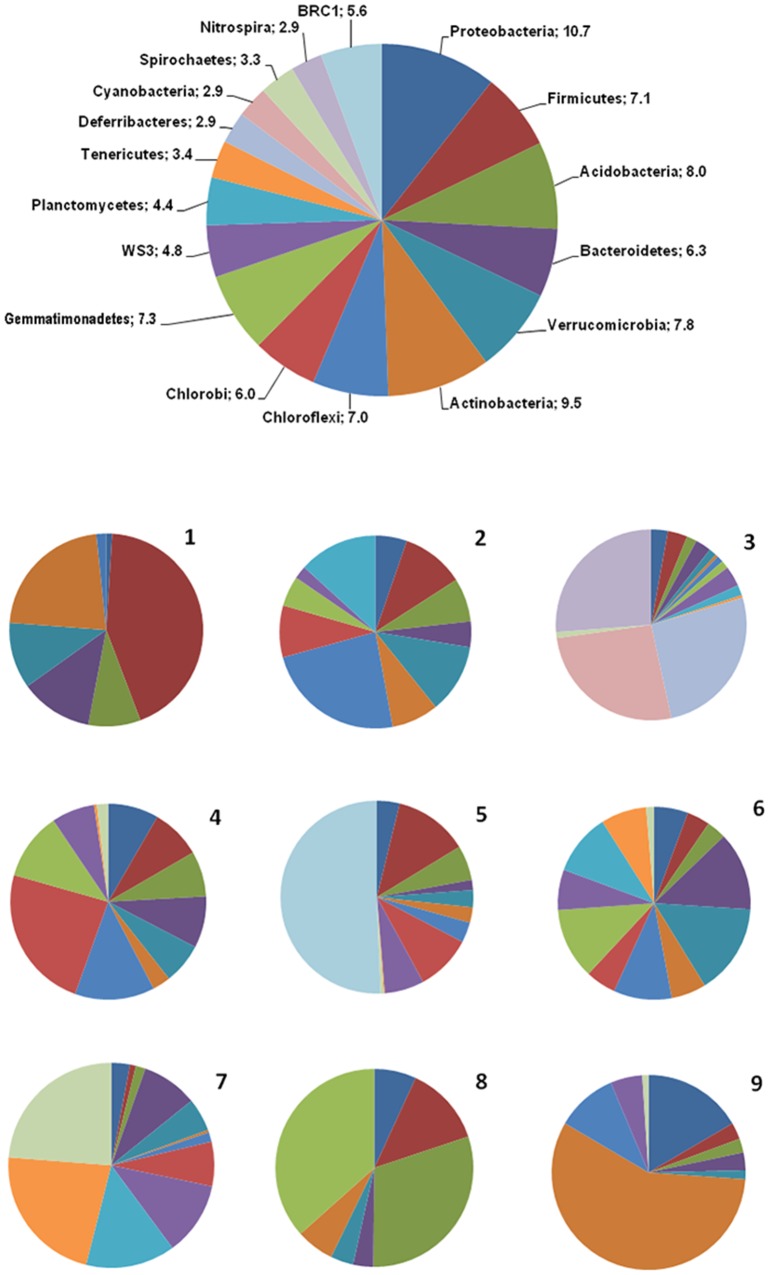
Piecharts based on the abundance (%) of bacterial phyla. Every pie shows the percent of the abundance (%) of the bacteria phyla.

The percentages of the different bacterial phyla present in each soil site, and the percentages observed in the whole study site (values consisting of an average of what obtained in the nine sites) are reported in Figure 8, where, for each site, a pie-plot is showed, representing the bacterial community as emerged by the pyrosequencing analysis of the V2–V3 16S rRNA bacterial gene region; each slice of the pie represents a bacterial phyla. The large pie represents the average of the entire microbial community of the saline soil, and shows the labels for each slice/bacterial phyla that are also valid for the pies of each site. At a glance, if one compares the average composition of the saline soil under study and the composition of the bacterial community in each sampled site, a considerable spatial variability is noted. What is visually evident from Figure 8 is the way the microbial communities differ greatly both qualitatively and quantitatively from site to site, although the sites are only fifty meters away. Although some groups (like *Proteobacteria* and *Actinobacteria*) were dominant in almost all the sites, a very different composition of the microbial communities among the sites appeared. The graphs also show, from site to site, the relationships among the major microbial groups, and that those who dominate in one site can be absent or just visible in the next one. In each site, given the spatial proximity, it is likely that certain species of bacteria prevail better than others, but this likelihood is apparently expressed in a different way from point to point, probably depending on local environmental forces, and also on positive and/or negative interactions between the different microbial groups.

## Discussion

### Relative abundances of the phyla found in soils naturally affected by salt, and spatial variability

Some of the bacterial phyla found in the salt-affected soil under examination were also reported by Ma and Gong [12]. Using meta-analysis, these authors retrieved 10,082 sequences longer than 250 bp from the two databases GenBank and RDP, using the search terms “saline” OR “hyper saline”, AND “soil” AND “16S”. Ma and Gong [12] reported that 90% of the bacterial sequences they enumerated belonged to six phyla, namely *Proteobacteria*, *Actinobacteria*, *Firmicutes*, *Acidobacteria*, *Bacteroidetes*, and *Chloroflexi*, which were also detected in our study.

All the bacterial phyla reported as “salinity related” in previous studies were enumerated in the present survey, and, in addition, some phyla that, according to different authors [15], [22], cannot be classified as “salinity related” were also found. This wide group contains the following phyla: *Nitrospira, Deferribacteres, Cyanobacteria/Chloroplast, Gemmatimonadetes, Planctomycetes, BRC1, Verrucomicrobia, Tenericutes, Spirochaetes, WS3* and *Chlorobi*. What is more, the following bacterial phyla were significantly related to soil salinity for the first time: *Nitrospira, Deferribacteres, Cyanobacteria/Chloroplast, Tenericutes* and *Spirochaete.,* These phyla were abundant in the two sites showing the highest salinity grade (sites 7 and 3). On the other hand, in the present survey, some phyla classified as “salinity related” by some authors [12] showed an equal distribution all over the sites, apparently uninfluenced by the degree of salinity.

*Proteobacteria* seemed to be one of the most common bacterial taxon in saline soils [12], [49]. In the present study, the occurrence of *Proteobacteria* is actually the highest, with 95.95% of frequencies in the sites, followed by the *Actinobacteria* that represented the second most spread taxon, as it was present in 83.39% of the sites. In contrast with what reported by other meta-analysis studies [12], [50], the third largest phylum was *Acidobacteria* (72.12%), followed by *Verrucomicrobia* (70.60%), *Gemmatimonodates* (66.14%), *Firmicutes* (64.14%), *Chloroflexi* (62.69%), *Bacteroidetes* (56.62%), and *Chlorobi* (54.09%). In addition to the nine phyla reported above, 8 phyla characterised by a relative patchy abundance were observed. These taxa with a leopard-spot distribution among the sites had been related to hypersaline soil [12], [13], [17], [23], [24], [25], [27] by other authors. This is the case of groups like *Cyanobacteria* and *Deferribacteres* which characterised site 3, or the *BRC1* group which represented an isolated “spot” in the study area, being present just in site 5. These findings indicated that testing the variability of microbial community in a saline soil using a spatial scale was a successful operation, since it was effective in providing a picture of the subdivision of the microbial community according to a micro-environmental gradient. Although we examined bacterial communities at coarse levels of taxonomic resolution, we expect that a distribution of the bacterial groups based on a mosaic-like scheme would also apply at finer levels of taxonomic resolution.

We noticed that *Proteobacteria* and *Actinobacteria* were common denominators among the investigated sites, being dominant in soils with both high and low salinity. Both phyla contain representatives of the most abundant halophilic bacteria occurring in saline soils [12]. The *Chloroflexi* group, which was found by some authors in hypersaline wastewater [12], [49], and is known to be a potential phototroph, was fairly well distributed between the different sites. *Firmicutes* can also be considered common denominators in all the nine sites, because they resulted homogeneously spread in the soil sites. This phylum was apparently absent in a number of hypersaline environments previously investigated [15], [46], but it was found to be abundant in low salinity environments such as deep-sea sediments [21]. Among the dominant genera assigned to *Firmicutes*, the *Bacillus* outstands, as it proves to be an important resource for exploring halophilic enzymes and metabolic pathways for pollutant remediation in saline soil [22].

The phyla *Gemmatimonodates* is known for having members showing active roles in biogeochemical transformations, especially in hypersaline soils, where it was undeservingly described as a minor phylum [26]. The assignments attributed to this phylum showed a patchy distribution in the studied area, with a relatively high abundance in site 8 and in other sites characterised by a low salinity level. Another phylum showing a discontinuous distribution was *Planctomycetes,* found in previous studies as symbionts of marine algae and sponges [16], [20]. *Nitrospira* dominated site 3 which was the site most affected by the salt crust coverage. Not surprisingly, *Nitrospira* phylum is represented by nitrite-oxidising bacterial species with a marked chemolithoautrophic nature. *Cyanobacteria* presented an identical distribution as that of *Nitrospira* and *Deferribactereres*, which were found as dominant groups in site 3. *Cyanobacteria* is a phylum represented by oxygen evolving and chlorophyll containing photosynthetic bacteria [17], [24], [25], [46]. While *Nitrospira* and *Deferribactereres,* have an important role in biogeochemical cycles, being ammonia-oxidising bacteria (AOB) in saline soils [23] and sulphur oxidising, respectively. The phylum *Deferribacteres* comprehends also chemoorganotrophic heterotrophs that breath anaerobically with terminal electron acceptors including Fe(II), Mn(IV), SO, Co(III), and nitrate [23]. The phylum *Verrucomicrobia* showed, on the one hand, a uniform distribution among the considered sites, with no correlation to salinity, but also, on the other hand, a highly significant dependence on organic matter, with a Spearman's rho value for C_org_ of 0.667, for p>0.01. The bacteria belonging to this group is likely to overcome the selection imposed by the high salinity by means of an intimate association with the organic matter of the soil, and perhaps with a direct involvement in the carbon cycle. *Spirochaetes* showed a particular distribution, as they were present with low abundance in almost all sites, but also showing a particular association with the site 7, where the phylum exhibited a very high abundance and a correlation with the presence of another bacterial phylum, the *Tenericutes*. *Spirochetes* are widely distributed in nature; presumably they play an important role as free-living microbes in environments such as soil. The *Spirochaeta* phylum also contains moderately halophilic bacteria, such as species of the *Halomonas* and *Deleya* genera, being members of the gamma subclass of *Proteobacteria* [28], [51]. Facultative aerobic halophilic *Spirochaeta* bacteria were isolated by various authors, close to salted lakes [18], [28], although the phylum had never been recovered from saline soils, even according to meta-analysis based studies. *Tenericutes* is a phylum of bacteria that contains the Class *Mollicutes* and that, as reported above, presented almost the same distribution of *Spirochaeta. Tenericutes* comprehend denitrifying bacteria, and, due to the lack of a cell wall, they are more sensitive to osmotic stress [28]. The correlation with *Spirochaetes* suggests a symbiotic or parasitic role of *Tenericutes* with respect to *Spirochaetes*.

A further interpretation key for understanding the relationship between the relative abundances of these bacterial phyla and salinity and the other soil properties, is given by the illuminating work done by Noah Fierer and colleagues [52] who suggested that certain bacterial phyla can be differentiated into copiotrophic and oligotrophic categories that correspond to the r- and K-selected categories used to describe the ecological attributes of plants and animals. By applying the copiotroph–oligotroph concept to saline soil phyla, we can further understand the structure and function of soil bacterial communities in extreme conditions, and in discontinuous/patchy environments. Copiotrophic bacteria that have higher growth rates, a greater degree of variability in population size, and lower substrate affinities than oligotrophic bacteria, should be dominating when there is abundance of nutritive substrates and, in general, a non-limiting situation. The oligotrophs should increase in relative abundance, as substrate quality and/or quantity declines over time and harsh environmental conditions prevail. Fierer et al. [52] found that bacteria belonging to the *Acidobacteria* phylum showed an oligotrophic behaviour while β-Proteobacteria, and *Bacteroidetes* exhibited copiotrophic attributes, changing their abundances in a predictable manner to changes in soil C availability. Moreover, Fierer et al. [52] found that, across 71 different samples, the *Acidobacteria* were less abundant, while the beta-Proteobacteria and the *Bacteroidetes* were more abundant. This study confirms the abundance of *Proteobacteria* all over the sites, and also shows a considerable presence of *Actinobacteria* as the second most spread taxon, and of *Acidobacteria* as the third largest phylum. Both *Acidobacteria* and *Actinobacteria* frequencies were not correlated to soil organic carbon contents (C_org_), while a strong dependence to C_org_ is exhibited by *Verrucomicrobia* and *Chlorobi* phyla.

## Conclusions

In the latest years, saline soils received a great attention because of the general shortage of arable land, and of the increasing demand for ecological restoration of areas affected by secondary salinisation processes. This is due to the fact that naturally salt-affected soils have a biotechnological potential in their microbial communities, which represent not only a gene reserve for future exploitation in biotechnological applications, assuming they could be used in some kind of restoration or conservation techniques of saline environments, but they can also serve as model systems for exploring the relationships between diversity and activity at the soil level in selective/limiting situations. As outlined in the introduction, very few studies succeeded in addressing the beta diversity of the microbial species in soils, according to the different salt concentrations and, at a different scale, to bacterial taxa distribution in relation to salinity gradients [12].

Although some of the enumerated phyla related to saline soils have already been found by other authors, this study complements the limited information available on these extreme habitats by providing specific information on the type of distribution of different bacterial groups as a function of spatial gradients in salinity and pH. The analysis of bacterial 16S rRNA-based datasets obtained from a naturally saline soil revealed significant differences in bacterial community composition and diversity, along an increasing salinity level, which underlies a multi-scale spatial variability. What is more, a spatial heterogeneity of microbial communities at a relatively small scale has emerged from this study, especially with respect to the macro-scale environmental scheme in terms of geography and soil. The soil of the study showed a patchy distribution of the vegetation structure and of chemical properties, which coincided with an heterogeneous distribution of many bacterial groups. Some bacterial phyla appeared, however, spread in the whole study area.

It is possible to make some assumptions that could be the basis for future in-depth studies on the association between groups of bacteria, or on their variance in certain extreme environments. The first assumption is that spatial autocorrelation in terms of microbial diversity can hardly be found at the soil scales used for physical-chemical studies. According to some authors [53], spatial autocorrelation in soil ranges from 30 cm to more than 6 m, depending on the sampling extent considered. In some locations, Franklin et al. [53] found up to four different correlation length scales. The presence of nested scales of variability suggests that the environmental factors regulating the development of the communities in the saline soil of the present study may have operated at different scales. The presence of spatial patterns in the distribution of bacteria was demonstrated at the microscale by Nunan et al. [54] who showed ranges of spatial autocorrelation of 1 mm and below. The second assumption is that an environment in which some limiting factors favour some microbial groups and not others is in fact compared to a set of islands that allow the formation of different communities, separated from each other by the discontinuity of the chemical-physical factors and by the availability of nutrients. One could imagine that in spite of the same element of “noise” (salinity), the spatial discontinuity allows the formation of more possible microbial assortments. Therefore, a patchy saline environment can contain not just a single microbial community selected to withstand extreme osmotic phenomena, but many different though efficient communities.The occurrence of a significant number of “salinity unrelated” phyla (e.g. *Nitrospira*, *Spirochaetes*) captures our interest, therefore we strongly believe a further analysis, and a further step in metatrascriptomic of functional genes, are needed.

Responding to the initial question on the role of salt concentration in defining the diversity of the bacterial community in a saline soil, we can say that salinity had the strongest effect on bacterial community structure, as revealed by the study of the correlation between soil properties and bacterial phyla occurrence. Soil pH and other chemical properties seemed to have a minor impact on bacterial group distribution when analysed at the considered spatial scale. The relative abundances of a number of taxonomic groups, as a matter of fact, changed significantly between soil sites according to differences in soil salt content. Nevertheless, the abundance of some other taxa resulted almost unaffected by the salinity level (e.g. *BRC1*, *Gemmatimonodates*). This may indicate, on the one hand, a high plasticity of bacterial phyla that evidently possess genera and species adaptable to different conditions, while on the other hand that the sensitivity to salinity of some groups is poor or, in any case, less dependent on other factors, such as the presence of organic matter, plant roots, etc.

Furthermore, it is not certain that bacterial phyla co-occurring at a given site occupy the same ecological niche; rather, the spatial variability can indicate the existence of different scales in the distribution of some major environmental factors, just as the salinity factor. In any case it is evident that the correlation of some groups (*Nitrospira*, *Deferribacteres*, *Spirochaetes*) to the degree of salinity seems to be a necessary condition for the proliferation of the species belonging to those particular groupings.

In conclusion, we feel the need to deepen the scale at which we analyse the bacterial communities in extreme environments. To go back to the more general discussion on saline system ecology, and to the measurement of the “extent of species replacement or biotic change along environmental gradients”, which corresponds to the beta diversity *sensu* Whittaker [55], one should distinguish between two rather antithetical phenomena: nestedness and turnover. In the saline soil here studied, we have seen that nestedness occurred only for some taxa, when the biota of a site with a lower number of representatives was a subset of a biota with a greater number of elements of the same taxa (i.e. *Bacteroidetes*, *Chloroflexi*, *Chlorobi*, *Gemmatomonadates*). In this case, the dissimilarity between two sites is related to the difference in specific *richness*, and it occurs even in the absence of a real turnover of species. In contrast, the spatial turnover implies that he replacement of some species by others can easily occur in a mutable environment, where rain and water movements can strongly change the distribution of salts, although it requires a different experimental scheme, with time-related samplings.

It appears evident that the assortment and distribution of microorganisms in a heavily fragmented environment depend on very complex dynamics of colonisation and dispersion, and that the analysis of the correlation between the population of microorganisms and environmental parameters, such as the organic matter, pH, and salinity, adds important information that can help to unravel the mechanisms of formation and structure of the bacterial communities.

## Supporting Information

Figure S1 **Location of the study area.**
(TIF)

Figure S2 **Sampling scheme.**
(TIF)

Figure S3 **Sequences lost during “quality refinement” steps.** The piecharts report the fraction of the sequences maintained and the fraction of sequences lost during quality refinement steps. The green portion of each piechart is the maintained portion of sequences (approximately more than 90% of the total sequences) while the other two portions (the red and the blue ones) are the portion of sequences lost during the trimming and the chimera check steps, respectively.
(TIFF)

Figure S4 **Nucleotide relative frequency distribution along the sequences.** The first 10 bases of each sequences file showed an unbalanced nucleotide distribution.
(TIF)
