# Correction: Salinity and Bacterial Diversity: To What Extent Does the Concentration of Salt Affect the Bacterial Community in a Saline Soil?

**DOI:** 10.1371/journal.pone.0114658

**Published:** 2014-11-25

**Authors:** The PLOS ONE Staff

Three authors were omitted from the author contributions section in the “contributed to the writing of the manuscript” sub-section. Please refer to the correct author contributions here:

Conceived and designed the experiments: LC GB AB. Performed the experiments: LC GB GLP. Analyzed the data: LC GB FP GLP. Contributed reagents/materials/analysis tools: CD AB GLP. Contributed to the writing of the manuscript: LC FP GB.GLP CD AB
