# Supplementary figures and images for: Salinity and Bacterial Diversity: To What Extent Does the Concentration of Salt Affect the Bacterial Community in a Saline Soil?
Source: PLoS One. 2014 Sep 4;9(9):e106662. doi: 10.1371/journal.pone.0106662 (PMC4154724; doi:10.1371/journal.pone.0106662)

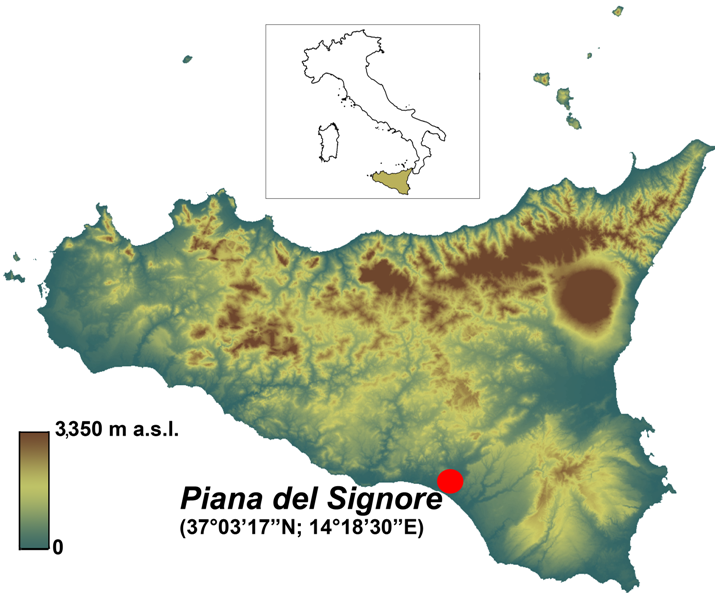

Supplement: Figure S1 — Location of the study area. (TIF) [file pone.0106662.s001.tif]

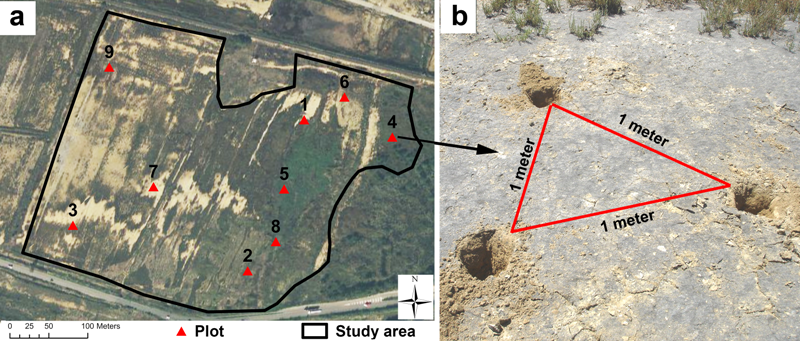

Supplement: Figure S2 — Sampling scheme. (TIF) [file pone.0106662.s002.tif]

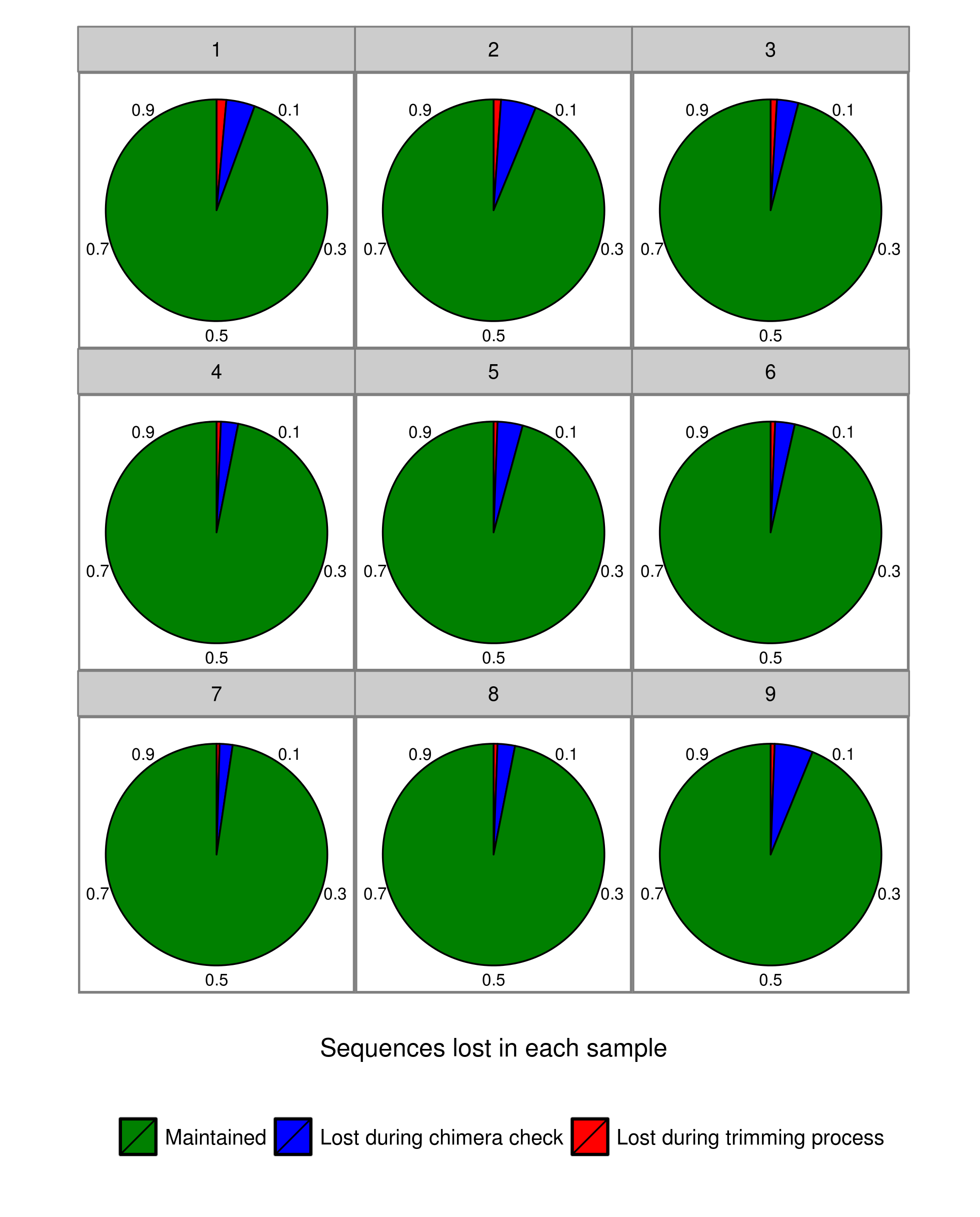

Supplement: Figure S3 — Sequences lost during “quality refinement” steps. The piecharts report the fraction of the sequences maintained and the fraction of sequences lost during quality refinement steps. The green portion of each piechart is the maintained portion of sequences (approximately more than 90% of the total sequences) while the other two portions (the red and the blue ones) are the portion of sequences lost during the trimming and the chimera check steps, respectively. (TIFF) [file pone.0106662.s003.tiff]

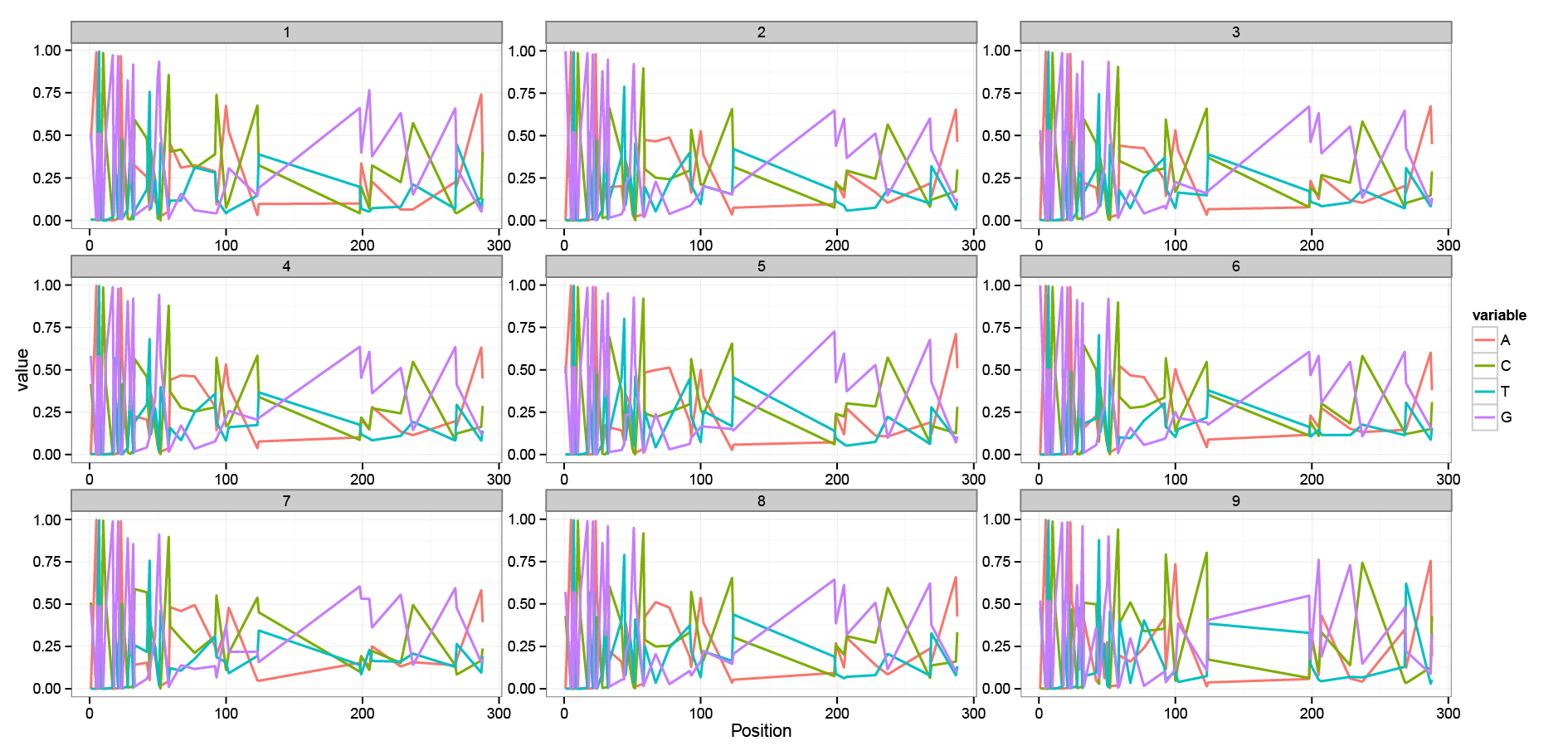

Supplement: Figure S4 — Nucleotide relative frequency distribution along the sequences. The first 10 bases of each sequences file showed an unbalanced nucleotide distribution. (TIF) [file pone.0106662.s004.tif]
